# The Role of Nutrition in Cancer Prevention - Should You Listen to Your Doctor or Influencer?

**DOI:** 10.1177/15598276221112331

**Published:** 2022-07-06

**Authors:** Shireen Kassam, Laura Freeman

**Affiliations:** 111990 King’s College Hospital, London; University of Winchester, Winchester, UK (SK); and Plant-Based Health Online, London, UK (LF)

**Keywords:** diet, nutrition, lifestyle, cancer, cancer prevention

## Abstract

A significant proportion of cancers could be prevented by adopting healthy lifestyle behaviours. In addition, healthy lifestyle factors can have a positive impact on cancer outcomes and survival. Yet, most physicians, including oncologists, do not dedicate a significant amount of time addressing these factors with their patients, who instead look to mainstream media and other non-medical sources for information. This has led to an increase in the number of ‘influencers’ in the wellness space who can accumulate a large and captive audience. At times, this has caused friction amongst healthcare professionals who feel that ‘influencers’ may overstate the potential benefits. The reality is that most people, physicians and the public alike, fail to recognise the immense power that lifestyle interventions hold. Rather than shy away from addressing these issues, we should be empowering our patients to take back control over their health. Here, we provide a personal perspective on why addressing lifestyle factors within cancer care is so important and that we can indeed work together with ‘influencers’ to amplify the message.

“With dietary risk factors being the leading cause of chronic illness and premature death, we need to take every opportunity to discuss this with our patients.”

A recent video by The Happy Pear on social media, now withdrawn, caused a massive media backlash from health professionals.^1^ The details of the video are less important only that it highlighted foods and other factors (such as exercise) that have been associated with a lower risk of breast cancer. Those living with a diagnosis of breast cancer felt the video blamed them for their cancer diagnosis and many health professionals felt the facts shared overstated the benefits of a healthy diet. The Happy Pear are two Irish brothers with a large following. They are vegan and advocate for a healthy plant-based diet through their recipes, books and courses, so of course many feel the information they provide is biased.

The 30-second video itself should not really have been so controversial but the mention of specific foods (mushrooms and soya) for reducing the risk of breast cancer was highly emotive for some. This is despite the fact that reputable cancer institutions share similar information on their websites.^2^ The video lacked nuance and we fully accept that neither a single food nor specific diet is going to eliminate a person’s risk of cancer. The overall quality of the diet is most important, with a focus on an abundance of minimally processed plant foods. We also acknowledge that at times social media ‘influencers’ may sensationalise or overstate benefits to attract followers. Nonetheless, this episode will undoubtably have made doctors feel reticent about broaching the topic of diet and lifestyle with their patients who have cancer. It is quick and easy to tell patients to ‘eat a healthy, balanced diet’ and ‘everything in moderation’, which of course can be interpreted in different ways and means many patients remain confused and either do not address their dietary habits or look to ‘influencers’ such as the Happy Pear, for dietary advice.

Here, we provide 2 perspectives, that of a doctor caring for people with cancer (Shireen) and a patient, also a doctor, who has had a diagnosis of cancer (Laura).

## The Doctor Perspective

We cannot ignore the fact that unhealthy diets are the leading cause of chronic ill health. Unhealthy diets are responsible for 1 in 4 deaths globally and a third of premature deaths in Europe and the United States.^3^ Around 1 million cancer deaths per year are due to a poor-quality diet with 20 million years lost in disability and premature death.^4^ Typically, unhealthy diets are too high in sodium, processed and unprocessed red meat (a Group 1 and 2a carcinogen, respectively) and insufficient in healthy plant foods and fibre. In the UK, this amounts to an estimated 18.8 deaths per 100,000 population from cancer due to dietary risk factors. Cancer Research UK states that healthier diets could prevent 1 in 20 cancers, that is, 18,750 new diagnoses per year or 50 new cases per day in the UK.^5^ Similar data exist for the United States.^6^ Yet when surveyed, the public are unaware of the extent of the impact of diet and lifestyle factors on cancer risk, with less than 50% being aware that processed red meat is a direct cause of cancer and that fruit, vegetables and fibre are protective.^7^ To shy away from the opportunity to discuss the impact of diet on cancer with my patients is therefore a hugely missed opportunity.

Of course, it’s not only about cancer risk. My patients often have several comorbidities that impact the treatments that are available to them and may also increase the side-effects of treatments offered. We know that underlying comorbidities not only increase the risk of cancer but that a cancer diagnosis itself increases the risk of cardiovascular disease and type 2 diabetes.^8^ Data from the UK biobank study showed that healthy diet and lifestyle habits reduced the risk of cardiovascular disease by 44% and type 2 diabetes by 38% in people with a diagnosis of cancer.^9^ In a study of 1.5 million cancer survivors from the US, the same risk factors that led to the initial cancer, namely obesity and smoking, were shown to increase the risk of a second cancer.^10^

Regardless of one’s diet choice, we cannot ignore the fact that meat-free diets, which emphasise the consumption of fruit, vegetables, whole grains and beans, do in fact significantly reduce the risk of cancer with vegetarians^11^ and vegans^12^ reducing their risk by around 15%. One reason for this reduction in risk may be that a healthy plant-based diet that also minimises the consumption of ultra-processed foods, otherwise known as a whole food plant-based diet, is associated with a healthier body weight. Another reason may be related to a healthier gut microbiome, which significantly impacts the functioning of the immune system. It is also becoming clear that a healthier gut microbiome can improve the response to certain anti-cancer treatments^13^ and as such, in line with international guidelines for cancer prevention and survival,^14^ we should be supporting our patients to adopt a diet that emphasises a variety of plant foods and provides adequate amounts of fibre.

Of course, most studies report relative risks, which sound much more dramatic than the impact on absolute risk. For example, when considering the impact of processed red meat consumption, a group 1 carcinogen, on colorectal cancer risk, the original study from 2015 reported that a 50-g portion of processed meat eaten daily increases the relative risk of colorectal cancer by 18%.^15^ For the individual that equates to an increase in lifetime risk from 5.6% to 6.6%, that is, a 1% increase in absolute risk, when comparing those who eat the least with those that eat the most processed red meat. This sounds rather less impressive and may be some would dismiss this as not being important to them. However, when considered at a population level, this amounts to 10 extra cases per 1000 population for those consuming the most processed red meat or 13% of all cases of colorectal cancer, equal to 5400 cases per year in the UK.^16^ In addition, there is an additive effect when addressing risk factors such that by consuming more fibre, avoiding alcohol, maintaining a healthy weight and undertaking regular physical activity, more than 50% of cases of colorectal cancer could be prevented.^17^ Optimising diet and lifestyle factors could prevent almost half of all cancers in high income countries.^14^ Similarly, the reduction in risk for developing other chronic conditions in those living with cancer are additive with a greater adherence to a healthy diet and lifestyle.^9^

We therefore need to find a sensitive way to address diet and lifestyle habits with our patients. My patients all ask me ‘What can *I* do?’ ‘What should I eat?’ If they do not, then I ask permission to discuss their diet and lifestyle and ask if they would be interested to learn more. Virtually, everyone at the point of a cancer diagnosis or at completion of treatment is open and receptive to this conversation. I recognise that for a variety of reasons patients differ in their ability to make and sustain healthy lifestyle habits. However, assuming a patient will be upset or will not want to change totally underestimated their abilities and misses an important teachable moment in an individual’s health journey.

## The Patient Perspective

I have been seeing patients in clinical practice for 16 years but my experience as a patient in 2016 changed my life – personally and professionally.

In my second pregnancy, my obstetrician found a lump in my neck at a routine 12-week appointment. A decision was made for watchful waiting but it was forgotten whilst I dealt with a number of antenatal and postnatal complications. Then, when my daughter was about 5 months old, I noticed the lump had changed and I made time to follow through with the necessary investigations. Shortly after, a diagnosis of thyroid cancer was made and I underwent a total thyroidectomy and neck dissection. I returned to work as a GP just 2 weeks later – not yet healed fully, physically or emotionally.

In the months that followed, I started my own research as how to reduce cancer risk as well as how to reduce cholesterol levels. My preoperative bloodwork had shown high cholesterol and my biopsy result a genetic mutation which predisposed to melanoma, breast and colon cancer. It didn’t take long to find the evidence for plant-based nutrition to accomplish both simultaneously. Having spent time feeling that my health was out of my hands, I now felt empowered with this new information (I had not learnt about nutrition in medical school or postgraduate training) and driven to make dietary changes that would support my future health. This sense of ownership and control has also allowed me to navigate ongoing scans and medical appointments more confidently.

Alongside a regular exercise regimen, a whole food plant-based diet has helped me to feel physically more energetic and provided a sense of control over my health. In a number of months, I started to translate this new knowledge on diet and lifestyle into clinical work with my patients – sharing and discussing the evidence with them wherever relevant and appropriate. I felt a heightened sense of connection with my patients – especially those living with cancer – and found that if approached sensitively and considerately, most were receptive to the message that they could optimise their health and wellbeing with food and lifestyle choices.

These discussions were never about blaming or shaming patients for their cancer diagnoses. Rather, it was – and still is – about sharing evidence-based information to empower them to make healthy choices, helping them and supporting them to understand that they themselves have an important part to play in their own journey. My experience is that many are open and motivated to use dietary changes, exercise and other healthy lifestyle habits as part of their treatment plan. At the same time, an honest approach is needed. We cannot of course, provide any health guarantees and it is important to ensure that patients are not oversold on the power of diet and lifestyle. Equally, I see it as my responsibility to show my patients how significantly diet and lifestyle choices can support their health alongside – not in place of – allopathic treatments.

Not all people living with cancer are able or ready for diet and lifestyle advice at the same stage in their cancer journey. Some might not be open to it at all. But all patients are owed, at the very least, accurate and actionable information as to how healthy diet and lifestyle choices can improve their health outcomes. As healthcare professionals, we have a moral duty to discuss this openly and compassionately and help patients living with cancer to the very best of our ability.

## Conclusions

With dietary risk factors being the leading cause of chronic illness and premature death, we need to take every opportunity to discuss this with our patients. Counselling on behaviour change requires a different skill set from the conventional allopathic approach to healthcare. This starts by educating ourselves as doctors and sharing this knowledge with colleagues a so we all take an evidence-based approach to dietary counselling. Incorporating this information a advice into the doctor–patient consultation is highly relevant for all aspects of clinical practice and can be hugely beneficial to people living with cancer.
